# MBD1/HDAC3-miR-5701-FGFR2 axis promotes the development of gastric cancer

**DOI:** 10.18632/aging.204190

**Published:** 2022-07-22

**Authors:** Changan Zhao, Jiyu Miao, Ruifang Sun, Rui Liang, Wenhu Chen, Yi Gao, Xiaofei Wang, Shuiping Han, Wenbao Zhao, Ting Lei, Chen Huang

**Affiliations:** 1 Department of Pathology, School of Basic Medical Sciences, Xi’an Jiaotong University Health Science Center, Xi’an 710061, Shaanxi Province, P.R. China; 2 Institute of Genetics and Developmental Biology, Translational Medicine Institute, Xi’an Jiaotong University, Xi’an 710061, Shaanxi Province, P.R. China; 3 Department of Hematology, The Second Affiliated Hospital of Xi’an Jiaotong University, Xi’an 710000, Shaanxi Province, P.R. China; 4 Department of Hepatobiliary Chest Surgery, Shaanxi Provincial Corps Hospital of Chinese People’s Armed Police Force, Xi’an 710054, Shaanxi Province, P.R. China; 5 School of Basic Medical Sciences and Forensic Medicine, Hangzhou Medical College, Hangzhou 310053, Zhejiang Province, P.R. China; 6 Department of Cell Biology and Genetics, Medical School of Yan’an University, Yan’an 716000, Shaanxi Province, P.R. China; 7 Department of Cell Biology and Genetics, School of Basic Medical Sciences, Xi’an Jiaotong University, Xi’an 710061, Shaanxi Province, P.R. China

**Keywords:** epigenetics, tumorigenesis, miRNA, gastric cancer

## Abstract

Gastric cancer (GC) remains one of the leading causes of cancer-related deaths worldwide due to the lack of specific biomarkers for the early diagnosis and universal accepted therapy for advanced GC. Lower levels of miR-5701 were found in the GC tissue from the online sequencing data and confirmed in the GC tissues and GC cell lines. Overexpression of miR-5701 inhibited the proliferation and migration of GC cells and promoted the apoptosis of these cells. Bioinformatics analyses and luciferase assay showed that miR-5701 targeted FGFR2, which acted as an oncogene in GC. Nude mice with GC cells overexpressing miR-5701 exhibited smaller tumor sizes and less lung metastases. The miR-5701 expression was directly, transcriptionally inhibited by MBD1 together with HDAC3 by binding together to form a complex. Knocked down MBD1 or HDAC3 increased the miR-5701 expression. These results indicated the potential use of exogenously administered miR-5701 or agents that elevated endogenous miR-5701 to inhibit GC, improving the prognosis of patients with GC.

## INTRODUCTION

Gastric cancer (GC) is the fifth most common cancer and has become the third leading cause of cancer deaths globally [1]. The incidence of GC varies markedly with geography. Asian countries have considerably higher rates than Northern Europe and North America. Chinese patients alone account for 42% of Asian cases [2]. Surgical resection remains the preferred treatment for GC. For the early GC cases, the 5-year survival can exceed 90%, but for advanced cases, the 5-year survival rate remains below 20%. This scenario is changing slowly with individualized therapies, including neoadjuvant chemoradiotherapy, molecular-targeted therapy, and immunotherapy. However, GC remains one of the leading causes of cancer-related deaths probably because of the absence of molecular selection and predictive biomarker [3]. No universally accepted therapy for advanced GC is available.

More than 30% of mRNAs are direct targets of miRNAs, and these miRNAs can play roles as oncogenes or tumor suppressor genes during the development of human cancers. Many recent studies have indicated that miRNAs are involved in the pathogenesis of GC. We searched and analyzed the online miRNA sequencing data and found that the expression of miR-5701 is lower in GC than in normal gastric tissue. However, the roles of miR-5701 in GC are not clear. miR-5701 is located on chromosome 15q11.2, and its role was studied in cervical cancer cells as a tumor suppressor gene [4]. The expression of miR-5701 is upregulated in breast cancer cells [5], downregulated in hepatocellular carcinoma cells [6], and depleted in extracellular vehicles of prostate cancer [7].

The expression and function of miRNAs were regulated by various factors, and DNA methylation, histone modifications, and noncoding RNAs are frequently involved in gastric carcinogenesis [8]. Methyl-CpG-binding protein 1 (MBD1) is a primary reader of DNA methylation and recruits methylases, histone deacetylases to methylated DNA [9]. The role of MBD1 in tumor progression regulation has been reported in cancers of the pancreas [10], prostate [11], and leukemia [12]. MBD1 utilizes its MBD and TRD domains to recruit transcriptional repressor proteins and silence tumor suppressor genes [13]. Previous studies [12, 14] have demonstrated that MBD1 might interact with HDAC3, which epigenetically regulates gene expression by acetylating lysine residues in the histone tails [15]. With the use of bioinformatics analysis, we predicted miR-5701 as a candidate target of MBD1 and HDAC3. Methylated sites might exist in the promoter region of miR-5701.

Based on the bioinformatics data, we sought to test the hypothesis that miR-5701 plays antitumor roles, and its expression is controlled by MBD1 and HDAC3 in GC. In the current study, we investigated the expression and role of miR-5701 in GC tissues and cell lines. The results showed that the expression of miR-5701 was indeed lower in GC and was apparently correlated inversely with the malignant behavior of GC. Overexpression of miR-5701 inhibited the proliferation and metastasis of GC cells *in vivo*, indicating the therapeutic potential of miR-5701.

## RESULTS

### Bioinformatics analysis of miR-5701 in GC

**Figure 1:**
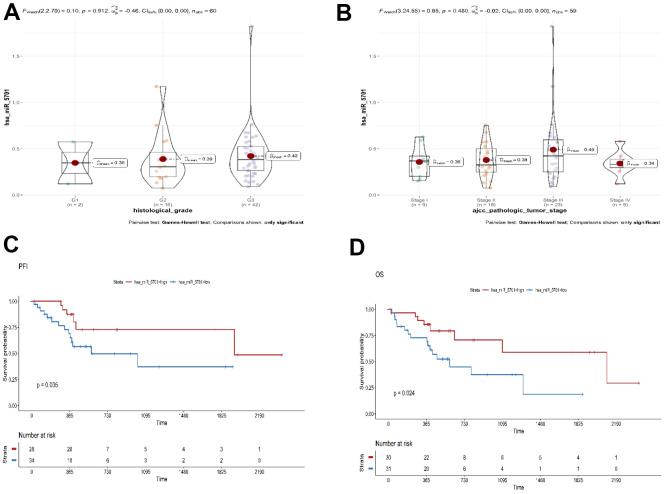
**Bioinformatics analysis of miR-5701 in GC.** (**A**, **B**) The expression of miR-5701 is associated with the pathological and histologic grade of GC. (**C**, **D**) Bioinformatics analyses were used to elucidate the effect of miR-5701 in GC tissues. The expression of miR-5701 was related to the PFI (progression-free interval event), OS (overall survival).

The TCGA database was used to elucidate the effect of miR-5701 in the GC tissues by bioinformatics approach. The expression of miR-5701 was lower in the GC tissues than in the healthy counterparts, and its expression was associated with the histologic and pathologic stages of GC (Figure 1A, 1B). The expression of miR-5701 was associated with progression-free interval (PFI, *P*=0.035), and overall survival (OS, *P*=0.024) in GC (Figure 1C, 1D), suggesting that miR-5701 played a key role as a tumor suppressor in GC.

### miR-5701 was downregulated in GC and acted as a tumor suppressor

**Figure 2:**
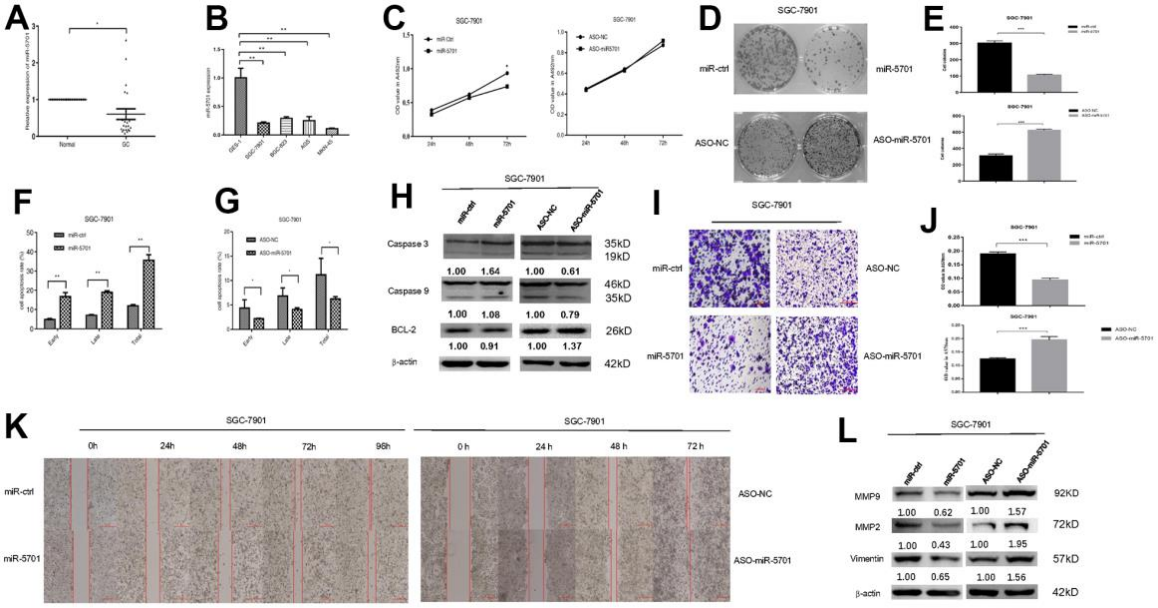
**Expression and function of miR-5701 in GC cell line SGC-7901.** (**A**) The expression of miR-5701 in GC tissues versus normal gastric tissues. (**B**) The expression of miR-5701 in GC cell lines. GES-1 was used as control. SGC-7901 cells were infected with miR-5701 or were treated with ASO-miR-5701. (**C**) Cell viability was measured by MTT assay at the time of 24h, 48h and 72h. (**D**, **E**) Cell proliferation was measured by colony formation assay; (**F**, **G**) Cell apoptosis was checked by flow cytometry; (**H**) Apoptosis-associated proteins were measured by Western blot; (**I**–**L**) Cell migration were measured by scratch assay and transwell assay, and migration associated proteins were measured by Western blot. Data are represented as the mean±SD and experiments were performed in triplicate; * p<0.05; ** p<0.01.

**Figure 3:**
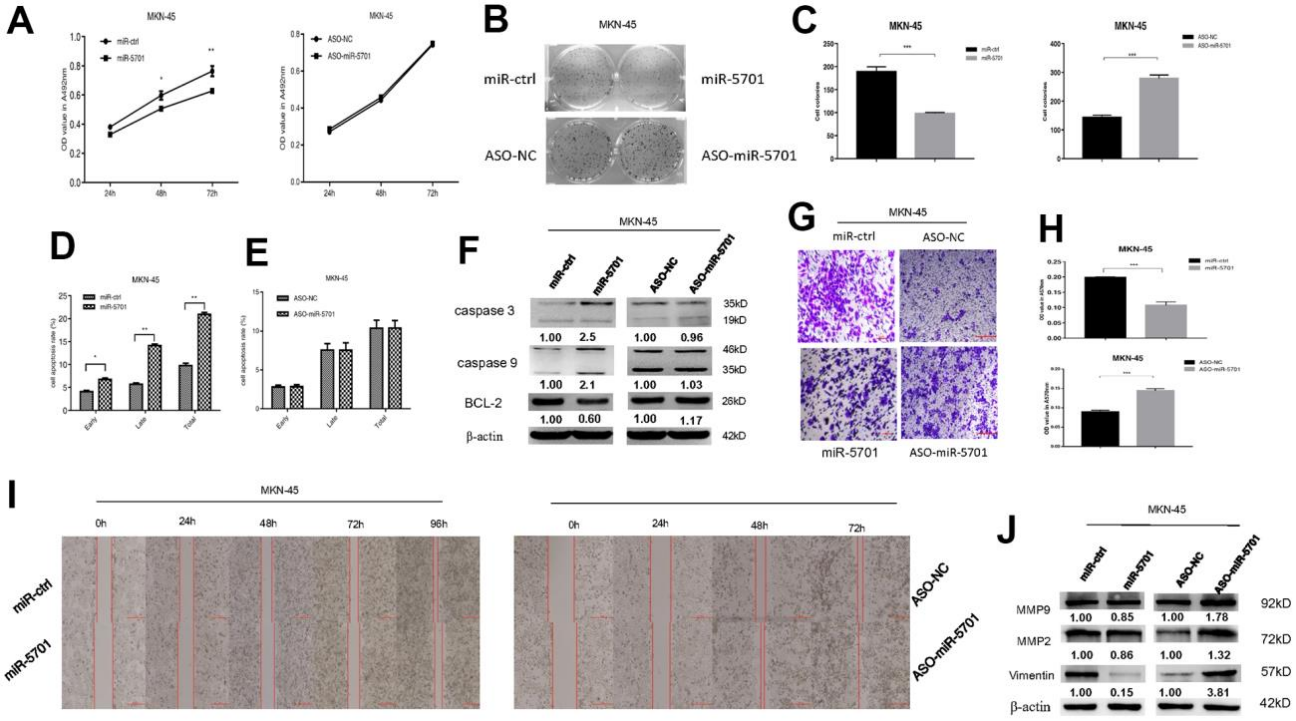
**Expression and function of miR-5701 in GC cell line MKN-45 which was infected with miR-5701 or were treated with ASO-miR-5701.** (**A**) Cell viability was measured by MTT assay at the time of 24h, 48h and 72h. (**B**, **C**) Cell proliferation was measured by colony formation assay; (**D**, **E**) Cell apoptosis was checked by flow cytometry; (**F**) Apoptosis-associated proteins were measured by Western blot; (**G**–**J**) Cell migration were measured by scratch assay and transwell assay, and migration associated proteins were measured by Western blot. Data are represented as the mean±SD and experiments were performed in triplicate; * p<0.05; ** p<0.01.

The miR-5701 expression in GC has not been reported yet. We first examined the levels of miR-5701 in the clinical GC samples and GC cell lines. Compared with the normal gastric tissues and normal gastric cell line, the expression levels of miR-5701 were significantly lower in the GC tissues and in several gastric cell lines (Figure 2A, 2B). To investigate the clinical significance of miR-5701 in GC, we divided patients with GC into high- and low-expression groups based on a previous PCR result. The results of the correlation analysis for the GC clinical features and miR-5701 expression are shown in the Table 1. We then tested the functions of miR-5701 in the GC cells. Overexpression of miR-5701 inhibited the cell viability of the GC cell line SGC-7901/MKN-45, while blockage of miR-5701 slightly promoted the cell viability in SGC-7901/MKN-45 cells (Figures 2C, 3A). Given that the basal level of miR-5701 in SGC-7901/MKN-45 was very low, blockage of miR-5701 just slightly promoted cell viability. Colony formation results showed that the overexpression of miR-5701 decreased the colony number, but blockage of miR-5701 increased the colony number of SGC-7901/MKN-45 (Figures 2D, 2E, 3B, 3C). Flow cytometry results also showed that overexpression of miR5701 promoted cell apoptosis, and blockage of miR5701 decreased apoptosis of SGC-7901/MKN-45 (Figures 2F, 2G, 3D, 3E), and apoptosis-associated proteins, such as caspase-3, caspase-9, and BCL-2, changed in the miR-5701 overexpressing cells and miR-5701 blocking cells (Figures 2H, 3F). Transwell and scratch results revealed that miR-5701 inhibited cell migration through the downregulation of migration-associated proteins, including MMP-2, MMP-9, and vimentin (Figures 2I–2L, 3G–3J). These results indicated that miR-5701 might be a tumor suppressor miRNA.

**Table 1:** Correlations of miR-5701 expression level with clinicopathologic features of GC.

**Characteristics**		**Number of cases**	**miR-5701 expression**		**P-value**
			**High**	**Low**	
Age(y)				
	≥60	16	5	11	0.9
	<60	12	4	8	
Gender				
	Male	22	8	14	0.04*
	Female	6	1	5	
Grade				
	G1+G2	8	3	5	0.50
	G3	20	5	15	
pTNM stage				
	I+II	4	1	3	0.86
	III+IV	24	7	17	
T phase				
	T1+T2	11	3	8	0.90
	T3+T4	17	5	12	
N phse				
	N0+N1	2	1	1	0.48
	N2+N3	26	7	19	

T tumor size, N regional lymph nodes status, GC gastric cancer.

*P<0.05 by the chi-square test.

### FGFR2 acted as a direct target of miR5701

**Figure 4:**
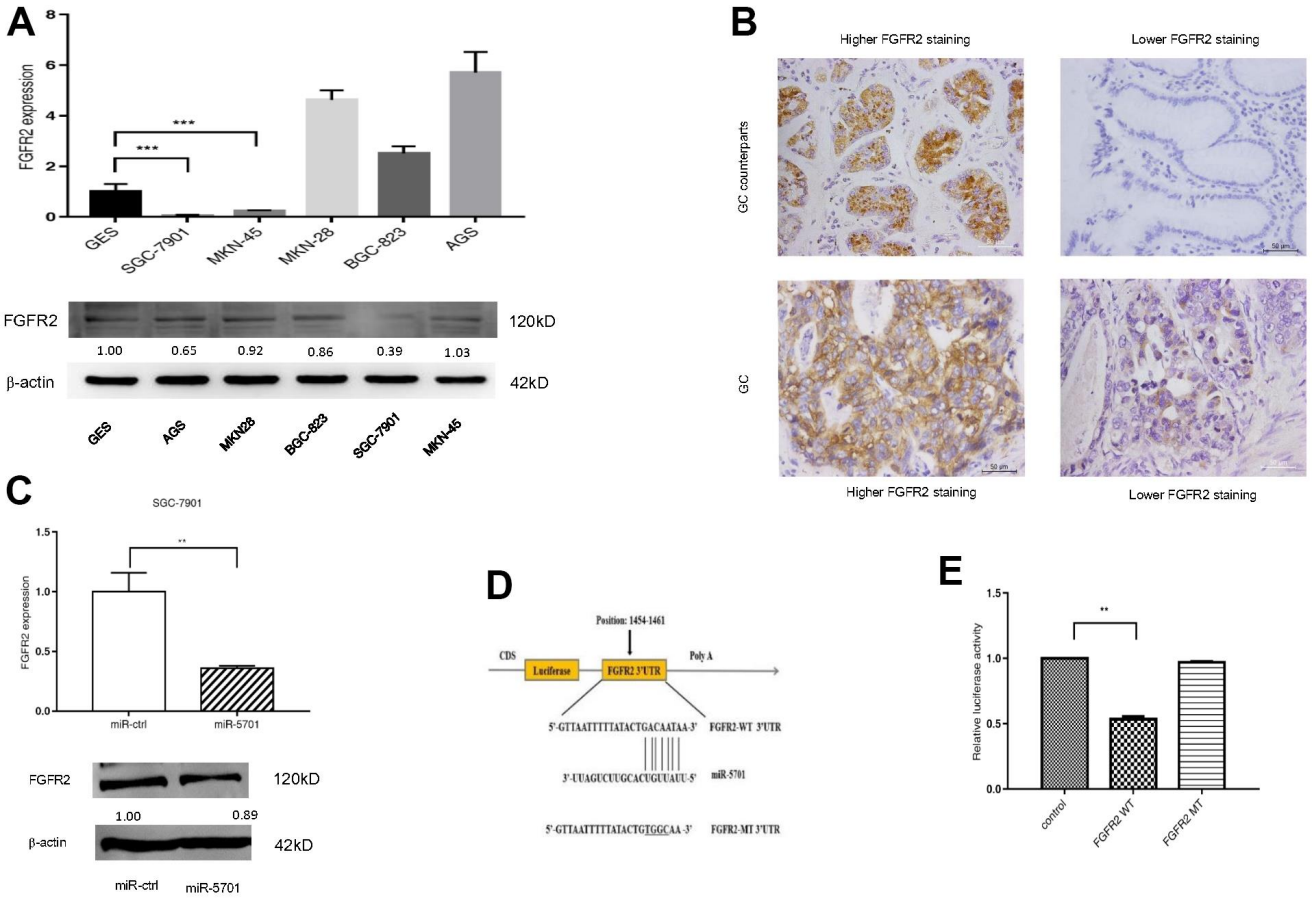
**FGFR2 is a direct target of miR-5701.** (**A**) The mRNA levels and protein levels in GC cell lines by qRT-PCR and Western blot. (**B**) Representative images of FGFR2 in GC tissues by immunohistochemistry. (**C**) mRNA level and protein level of FGFR2 after miR-5701 overexpression in SGC-7901. (**D**) miR-5701 is highly conserved across species and has binding sites within the 3′-UTR of human FGFR2; (**E**) Relative luciferase activity was measured to assess the target relationship of miR-5701 to FGFR2. Data are represented as the mean±SD and experiments were performed in triplicate; * p<0.05; ** p<0.01.

Using bioinformatics algorithms, we searched several databanks for potential miR-5701 target genes and found that FGFR2 may be a candidate target, because its 3’-UTR matched miR-5701 well. We then examined the expression levels of FGFR2 in several GC cell lines and in clinic GC tissues. The results showed that the expression levels of FGFR2 were uneven and also apparently not inversely correlated with miR-5701 (Figure 4A, 4B). Nevertheless, overexpression of miR5701 led to the downregulation of FGFR2 at the mRNA and protein levels (Figure 4C and Supplementary Figure 1). Furthermore, luciferase assay showed that miR-5701 suppressed the luciferase activity of the construct containing wild type 3’-UTR but not the construct containing mutant 3’-UTR of FGFR2 (Figure 4D, 4E). Data illustrated in Figure 4 suggested that FGFR2 functioned as a direct target of miR5701.

### FGFR2 functioned as an oncogene to promote GC proliferation and migration

**Figure 5:**
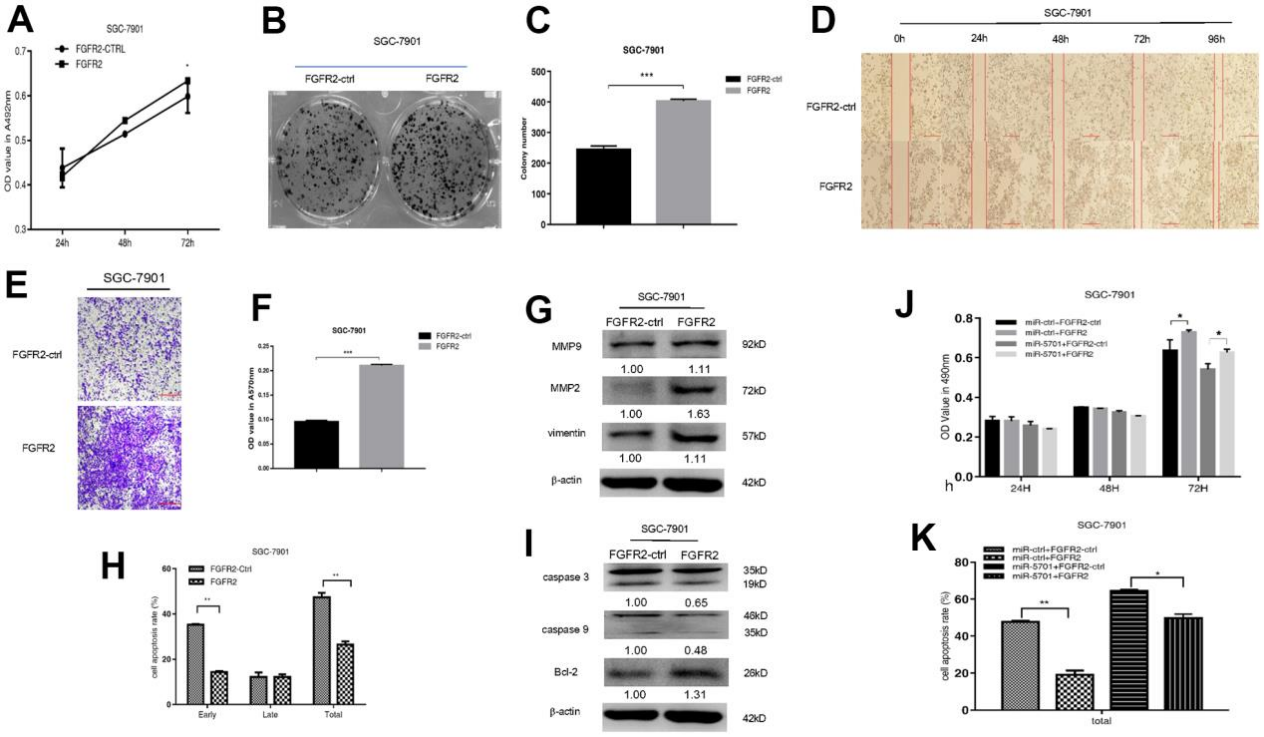
**FGFR2 functions in SGC-7901 cells.** SGC-7901 cells were transfected with FGFR2 overexpressed plasmid and (**A**) Cell viability was measured by MTT assay at the points of 24h, 48h and 72h; (**B**, **C**) Cell proliferation was measured by colony formation assay; (**D**–**G**) Cell migration was measured by transwell assay and scratch assay, and migration associated proteins were measured by western blot. (**H**, **I**) Cell apoptosis rates were measured by flow cytometry and apoptosis associated proteins were measured by Western blot. SGC-7901 cells were transfected with FGFR2 overexpressed plasmid or its control, at the same time were infected with miR-5701 or its control, and (**J**) Cell viability were measured by MTT assay at the points of 24h, 48h and 72h; (**K**) Cell apoptosis rates were measured by flow cytometry at 48h.

**Figure 6:**
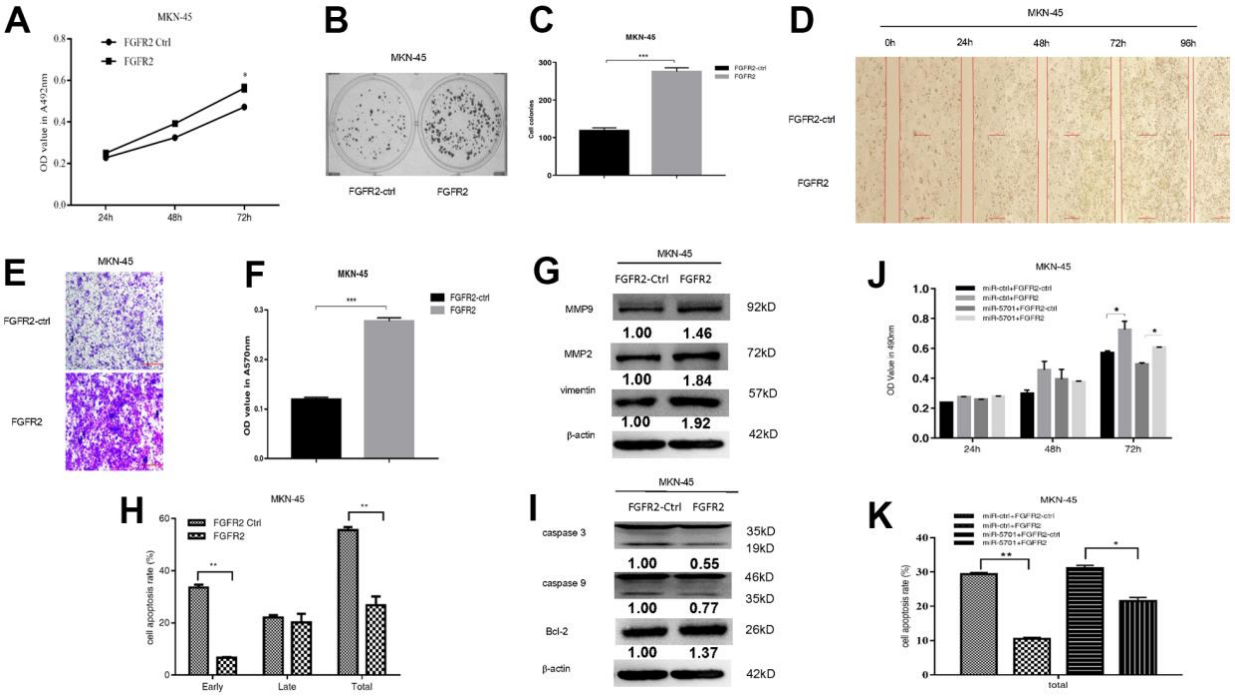
**FGFR2 functions in MKN-45 cells.** MKN-45 cells were transfected with FGFR2 overexpressed plasmid and (**A**) Cell viability was measured by MTT assay at the points of 24h, 48h and 72h; (**B**, **C**) Cell proliferation was measured by colony formation assay; (**D**–**G**) Cell migration was measured by transwell assay and scratch assay, and migration associated proteins were measured by western blot. (**H**, **I**) Cell apoptosis rates were measured by flow cytometry and apoptosis associated proteins were measured by Western blot. MKN-45 cells were transfected with FGFR2 overexpressed plasmid or its control, at the same time were infected with miR-5701 or its control, and (**J**) Cell viability were measured by MTT assay at the points of 24h, 48h and 72h; (**K**) Cell apoptosis rates were measured by flow cytometry at 48h.

FGFR2 is frequently amplified and overexpressed in GC, but the clinic pathological impacts of high FGFR2 expression are not consistent among studies [16]. We constructed the overexpression plasmid of FGFR2 and transfected it into the SGC-7901/MKN-45 cells. The result showed that FGFR2 overexpression promoted cell viability (Figures 5A, 6A), colony formation (Figures 5B, 5C, 6B, 6C), migration, and invasion (Figures 5D–5G, 6D–6G) by increasing migration-associated MMP2, MMP-9, and vimentin. This overexpression also simultaneously decreased cell apoptosis by inhibiting caspase-3 and caspase-9 and activating BCL-2 (Figures 5H–5L, 6H, 6I). These results suggested that FGFR2 functioned as an oncogene in GC. We simultaneously overexpressed miR-5701 and FGFR2 in the GC cells. The results showed that miR-5701 counteracted the functions of FGFR2, inhibited cell viability (Figures 5J, 6J) and increased cell apoptosis (Figures 5K, 6K) regulated by FGFR2. Thus, FGFR2 acted as an oncogene to promote GC cell proliferation and migration, while miR-5701 was an antimiR to inhibit GC cell proliferation by targeting FGFR2.

### miR-5701 inhibited GC cell proliferation *in vivo*

**Figure 7:**
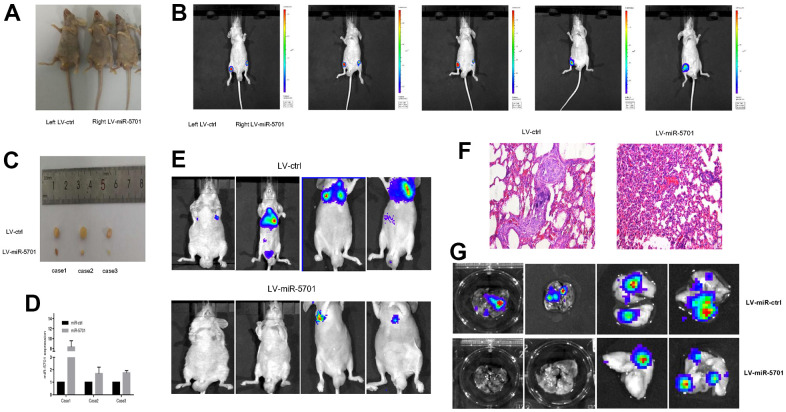
**miR-5701 inhibited GC proliferation and metastasis *in vivo*.** (**A**) SGC-7901 cells were infected with LV-miR-5701 or LV-ctrl and inoculated into groin subcutaneous tissue of nude mice, left for LV-ctrl, right for LV-miR-5701. (**B**) Tumors were measured by *in vivo* bioluminescence image. (**C**) Mice were sacrificed and tumors were isolated. (**D**) miR-5701 expressions in tumors were detected by qRT-PCR. (**E**–**G**) SGC-7901 cells were infected with LV-miR-5701 or LV-ctrl and injected into tail vein of nude mice, tumor metastasis in lung was detected by HE staining and by *in vivo* bioluminescence imaging.

Aforementioned results indicated that miR-5701 acted as an antimiR to inhibit GC cell proliferation and migration. We then constructed a lentivirus containing miR-5701 and infected the GC cells. We injected these cells into the right lateral inguinal region of nude mice (Figure 7A) to assess the tumor proliferation and metastasis. The left inguinal region was injected with GC cells infected with LV-control. After four weeks, the tumor growth was markedly inhibited by LV-miR-5701 compared with the LV-control (Figure 7B), and the sizes of the tumors were obviously smaller than those of the control (Figure 7C). The qRT-PCR results confirmed that the miR-5701 expression levels were increased in the xenograft tumors (Figure 7D). Meanwhile, we also injected GC cells overexpressing miR-5701 into the caudal vein of nude mice to assess the effect of miR-5701 on the metastasis of GC cells *in vivo*. Compared with the LV-control group, miR-5701 reduced lung metastasis (Figure 7E–7G). These results further confirmed *in vivo* that miR-5701 played antitumor roles in GC.

### miR-5701 was downregulated in GC cells related to MBD1 and HDAC3

**Figure 8:**
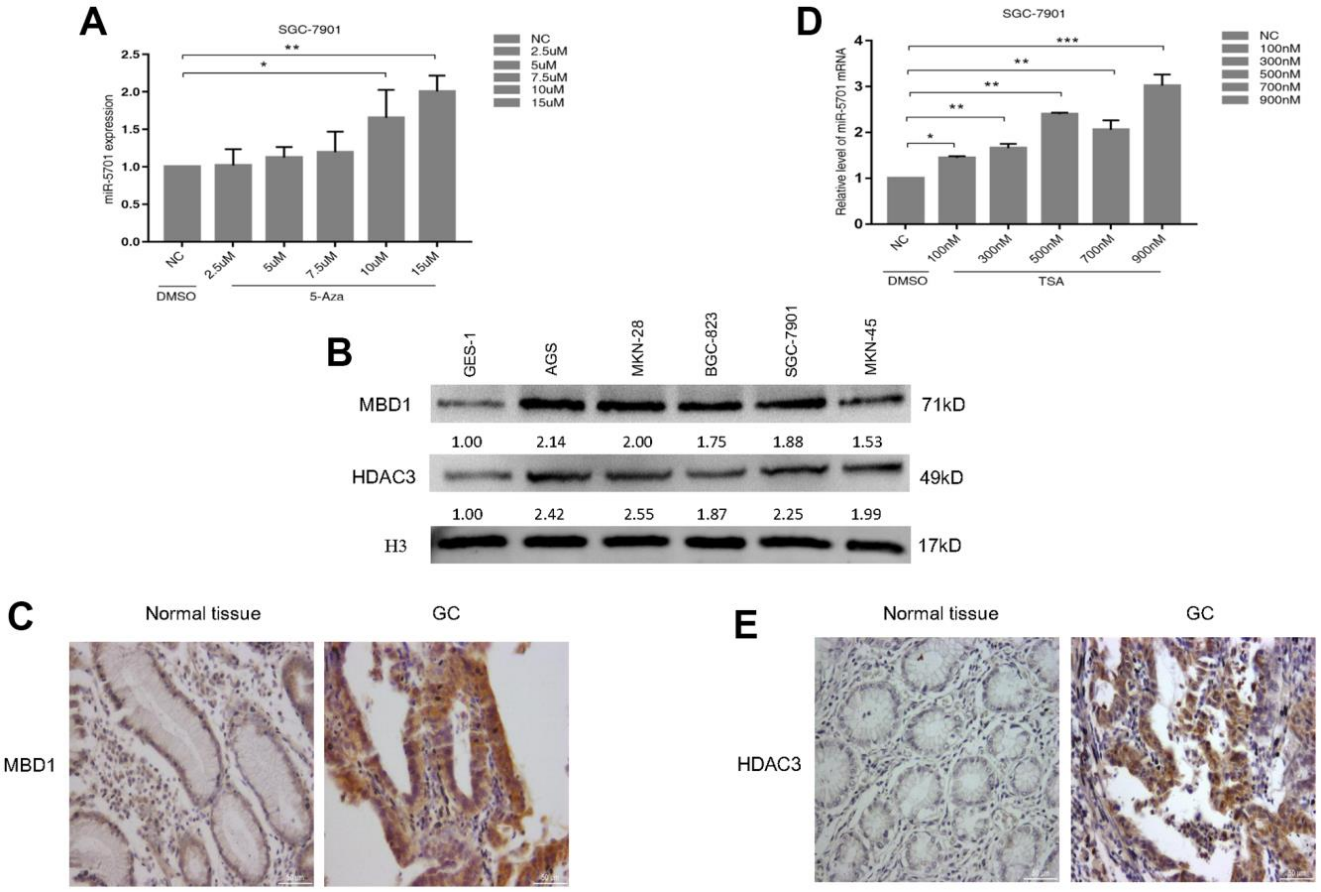
**miR-5701 expression was regulated by MBD1 and HDAC3.** (**A**) SGC-7901 cells were treated with 5-Aza at different concentration and miR-5701 expression was measured by qRT-PCR. (**B**) MBD/HDAC3 levels of several GC cell lines were measured by western blot. (**C**) The representative images of MBD1 in GC tissue versus normal tissue by immunohistochemistry. (**D**) SGC-7901 cells were treated with TSA at different concentration and miR-5701 expression was measured by qRT-PCR. (**E**) The representative images of HDAC3 in GC tissue versus normal tissue by immunohistochemistry.

miR-5701 played antitumor roles and was downregulated in GC, but its regulatory mechanism is unclear. We first examined the effects of DNA methylation inhibitor 5-azacytidine (5-Aza) on the miR-5701 expression in the GC cells. The results showed that the expression levels of miR-5701 increased in a dose-dependent manner after 5-Aza treatment (Figure 8A). We then checked the expression levels of methyl-CpG binding proteins (MBD1) in several GC cell lines and GC tissues. The data consistently showed that the protein levels of MBD1 were higher in the GC cell lines (Figure 8B) and GC tissues (Figure 8C) than in normal gastric cell line and gastric tissue. Furthermore, we examined the effects of the histone deacetylase inhibitor Trichostatin A (TSA) on the miR-5701 expression in the GC cells. We also found that TSA induced an increase in the miR-5701 expression in a dose-dependent manner (Figure 8D). Similarly, we examined the protein levels of histone deacetylase (HDAC3) in the GC cell lines and GC tissues. The data also showed that HDAC3 expression was higher in the GC cell lines (Figure 8B) and GC tissues (Figure 8E) than those in the normal gastric cell line and normal gastric tissue. These results indicated that miR-5701 expression was regulated by DNA methylation and acetylation.

**Figure 9:**
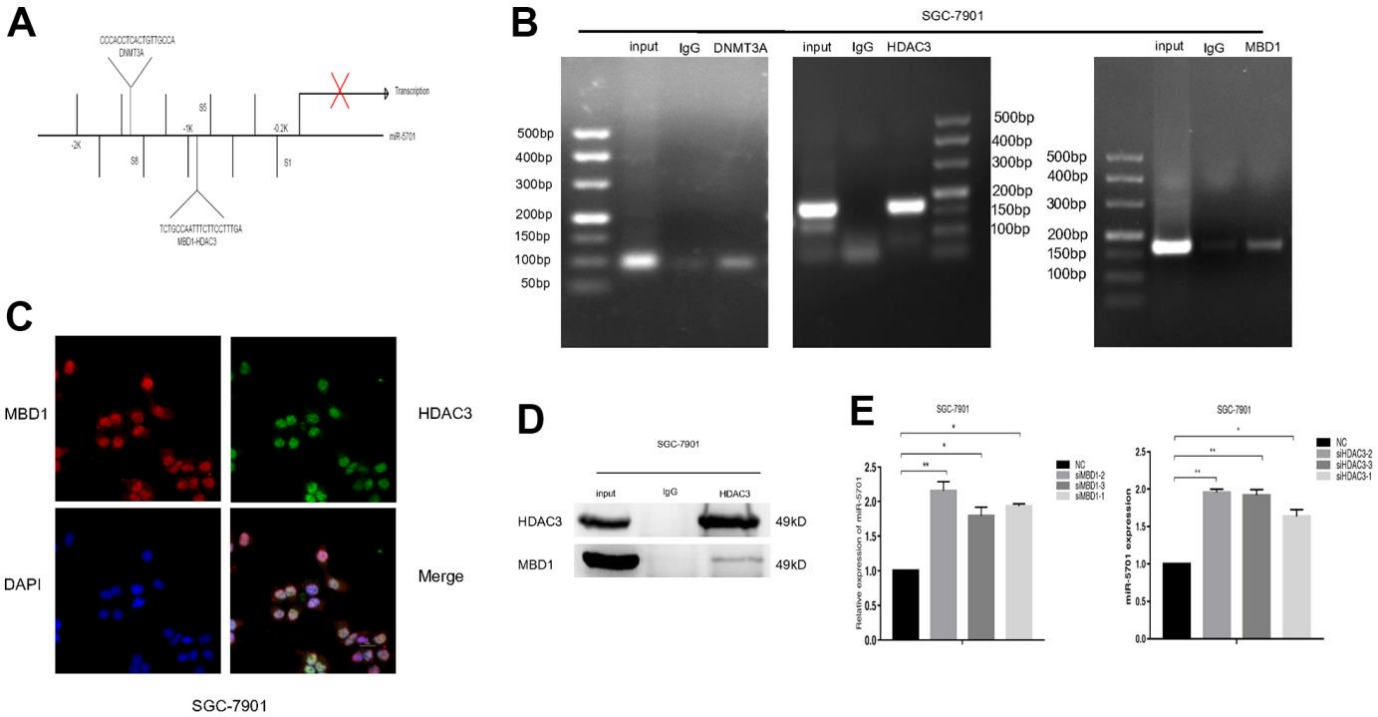
**MBD1 and HDAC3 directly bound to the promoter region and inhibited the expression of miR-5701.** (**A**) The binding sites of MBD1, HDAC3 and DNMT3A in the promoter of miR-5701. (**B**) ChIP-PCR showed that DNMA3A, HDAC3 and MBD1 directly bound to the promoter of miR-5701. (**C**) Immunofluorescence staining showed that MBD1 and HDAC3 co-localized in nuclei of SGC-7901 cells. (**D**) IP assay showed that MBD1 directly bound to HDAC3 at the protein level. (**E**) SGC-7901 cells were treated with siMBD1 or siHDAC3 and the expression of miR-5701 was measured by qRT-PCR. Data are represented as the mean±SD and experiments were performed in triplicate; *p<0.05, **p<0.01**.

To further understand whether MBD1 and HDAC3 were directly bound to the promoter of miR-5701, we designed several pairs of primers for the different regions of the miR-5701 promoter (Figure 9A) to perform chromatin immunoprecipitation (ChIP)-PCR. The results showed that DNMT3A, HDAC3, and MBD1 directly bound to the miR-5701 promoter in the GC cells (Figure 9B). Next, immunofluorescence staining showed that MBD1 and HDAC3 were colocalized in the nuclei of the GC cells (Figure 9C). Coimmunoprecipitation (CoIP) assay further indicated that HDAC3 and MBD1 formed a complex to control the expression of miR-5701 (Figure 9D). When we knocked down MBD1 or HDAC3, the expression of miR-5701 increased significantly (Figure 9E). These results demonstrated that the downregulated expression of miR-5701 in GC cells was related to the DNA hypermethylation and hyperacetylation.

**Figure 10:**
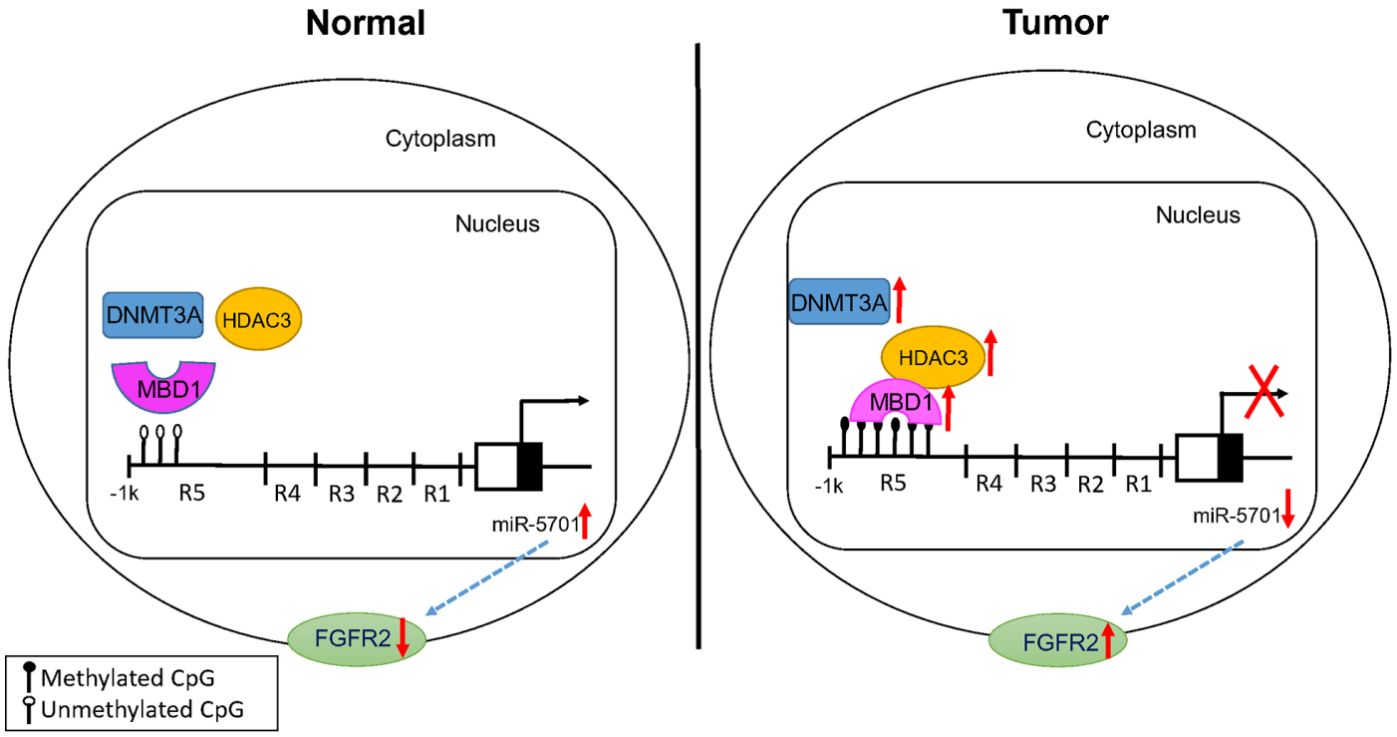
Proposed model for the effects of MBD1/HDAC3-miR-5701-FGFR2 axis in GC.

Based on the results, we propose a novel MBD1/HDAC3-miR-5701-FGFR2 signaling axis in human GC cells (Figure 10). We have demonstrated that MBD1 promoted the progression of gastric cancer by recruiting HDAC3 to form a complex, silencing the expression of anti-tumor miR-5701, and increasing the expression of the miR-5701 targets FGFR2. This novel MBD1/HDAC3-miR-5701-FGFR2 signaling axis may potentially serve as a new therapeutic target for GC.

## DISCUSSION

Data from the GC tissues, cultured GC cells, and tumor-bearing nude mice provide convincing evidence that low miR-5701 is associated with the progression of GC and that elevated level of miR5701 plays antitumor roles. Our data showed that miR-5701 directly targeted the 3’-UTR of FGFR2, and overexpression of FGFR2 promoted the proliferation and migration and inhibited the apoptosis of GC cells. Overexpression of miR-5701 could counteract the role of FGFR2 and inhibit the proliferation and metastasis of GC *in vitro* and *in vivo*. We further demonstrated that lower expression of miR-5701 in GC was associated with MBD1 and HDAC3 directly bound to its promoter region. Thus, our results indicated that miR-5701 played antitumor roles in GC and may be a potential therapeutic molecule.

Little information on miR-5701 in human cancers is available. The expression of miR-5701 was mentioned in several sequencing studies, and its levels varied in different human cancers [4–7]. miR-5701 functions as a tumor suppressor gene in cervical cancer [4], while its roles in other cancers remain ambiguous. Our data suggested that miR-5701 also played a role as a tumor suppressor gene in GC. We predicted and confirmed for the first time that FGFR2 was a direct target of miR-5701. The FGFR2 gene is located on human chromosome 10q26, which encodes FGFR2b and FGFR2c isoforms because of alternative splicing [17]. FGFR2 plays oncogenic or antioncogenic role in different cancers [17]**.** Aberration of the FGFR2 signaling pathway resulting from gene mutation, overexpression, amplification, or aberrant transcription regulation is related with the tumorigenesis and progression in cancers of the stomach [18], breast [19], bladder [20], and lung [21]. Our data consistently confirmed that FGFR2 functioned as an oncogene to promote GC progression, while miR-5701 expression was lower in GC than in normal gastric tissue. In addition, the overexpression of miR-5701 could counteract the effects caused by FGFR2 and inhibit GC progression *in vitro* and *in vivo*.

The mechanism of miR-5701 downregulation in GC remains uncertain, while epigenetic modifications, such as DNA methylation and histone acetylation, are major causes of gene silencing in cancer. Genome-wide analysis showed that many tumor suppressor genes have been found to be aberrantly hypermethylated in GC [22, 23]. MBD1 is a major protein that binds methylated CpG to function the silencing effect of DNA methylation, and MBD1 is involved in the tumorigenesis of many human cancers [24, 25]. MBD1 functions as an epigenetic regulator by different ways, including the formation of MCAF1/MBD1/SETDB1 complex or the MBD1-HDAC3 complex [26]. However, the role of MBD1 in GC still needs further elaboration, and whether lower expression of miR-5701 is related with MBD1 also needs to be demonstrated. Our results showed that MBD1 not only bound to methylated-CpG islands of miR-5701 but also recruited HDAC3 to form the MBD1-HDAC3 complex and negatively regulate the transcription of miR-5701. Consistently, treatment with 5-Aza or TSA or knockdown of MBD1 or HDAC3 upregulated the expression of miR-5701. HDAC3 has been illustrated to play a special role in the development of cancers, and recent studies have revealed that HDAC3 inhibit tumor suppressor genes, such as p53 [27], PUMA [28], DTWD1 [29], and FOXA2 [30], in GC. In this study, data from 5-Aza or TSA treatment, MBD1 or HDAC3 ChIP PCR, MBD1 or HDAC3 knock-down, and MBD1 IP showed that MBD1 together with HDAC3 bound to methyl-CpG to inhibit miR-5701 expression. However, whether HDAC3 alone inhibited miR-5701 expression through histone acetylation needs further investigation.

Therefore, our findings demonstrated that hypermethylation in GC led to the binding of MBD1 and HDAC3 and downregulation of miR-5701. Lower levels of miR-5701 in GC was coincident with the increased expression of FGFR2. The overexpression of miR-5701 in GC cells inhibited proliferation and migration *in vitro* and *in vivo*. Our results indicated the potential use of exogenous administered miR-5701 or agents that elevated endogenous miR-5701 to inhibit GC, improving the prognosis of patients with GC.

## MATERIALS AND METHODS

### GC tissue samples and cell lines

A total of 22 paired GC tissues and counterpart tissues were obtained from the patients who had no local or systemic treatment before surgery at the First Affiliated Hospital of Xi’an Jiaotong University. All tissues were checked by different professional pathologists. Informed consent was obtained from each patient and was approved by the Ethics Committee of Xi’an Jiaotong University. BGC823, SGC-7901, MKN-45, AGS, and normal gastric epithelium cell line (GES-1) were acquired from the Cell Bank of the Chinese Academy of Sciences. The cells were maintained in RPMI-1640 medium (Gibco-BRL, Grand Island, NY, USA) or DMEM (Gibco-BRL, Grand Island, NY, USA) supplemented with 10% FBS (Gibco) and kept at 37° C and in 5% CO_2_ environment. The cell line SGC-7901 was authenticated with STR analysis and without contamination by Hela cells.

### RNA extraction and qRT-PCR

Total RNA was extracted from the tissues and cells lines by using TRIzol reagent (Invitrogen, Carlsbad, CA, USA). The qRT-PCR was performed according to the methods as previously described [31]. All primers applied in this study are listed in the Supplementary Tables 1, 2.

### Plasmids, siRNA, and transfection

Pre-miR-5701 expression vectors and control vector were chemically synthesized in the pcDNA6.2-GW/EmGFP vector (Invitrogen, Carlsbad, CA, USA). The overexpressed plasmid of FGFR2, miR-5701 inhibitor, and all siRNA (i.e., siDNMT3A, siHDAC3, and siMBD1) were purchased from the GenePharma Corporation (SGC, Shanghai, China). The FGFR2 wild-type and mutated mRNA were cloned in between the SacI and XhoI sites of the pmirGLO expression vector (Invitrogen, Carlsbad, CA, USA). All related sequences used are listed in the Supplementary Table 2. The SGC-7901 cells were treated with 5-Aza (2.5, 5, 7.5, 10, and 15 μM; MedChemExpress, Shanghai, China) for 3 days and TSA (100, 300, 500, 700, and 900 nM; MedChemExpress, Shanghai, China) for 24 h. Then, the total RNA was extracted using the TRIzol protocol.

### Cell viability assay and apoptosis assay

MTT (Sigma, Germany) assay was performed to analyze the cell proliferation. MTT assay was performed based on the methods as previously described [32].

The cells were cultivated into six-well plates. At 48 h after transfection, the cells were labeled with the PI/FITC-Annexin V Apoptosis Detection Kit (7Sea Biotech, Shanghai, China), according to the manufacturer’s instructions. Apoptotic cells were checked using flow cytometry (Becton Dickinson, San Jose, CA, USA) and analyzed by Modfit software (Verity Software House, Augusta, ME, USA).

### Colony formation assays

SGC-7901/MKN-45 (2,000 cells/well) were seeded onto six-well plates at 24 h after transfection and cultured for 10 days. Cell colonies were fixed in 4% paraformaldehyde, stained with 1% crystal violet, and then photographed.

### Transwell assays

SGC-7901/MKN-45 (2×10^5^ cells/well) were seeded onto six-well plates. After transfection with overexpressed plasmids, the cells were harvested and counted. Then, 2×10^4^ cells were diluted in 200 μl of FBS-free medium and plated into the upper Transwell chambers (Millipore, Billerica, MA, USA), and the bottom wells of a 24-well plate were filled with 600 μl of complete medium. After 36 h of incubation, cells that did not invade through the membrane of the Transwell chamber were carefully removed. The invaded cells were stained with 1% crystal violet.

### Tumorigenicity assays in nude mice

The 4-week-old male nude mice were acquired from the Animal Center of Xi’an Jiaotong University and fed under aseptic condition. The SGC-7901 cells were infected with LV-miR-ctrl or LV-miR-5701. Then, 1×10^7^ infected cells were injected subcutaneously into both lateral inguinals of nude mice. The same amount of infected SGC-7901 cells were simultaneously injected into the caudal vein of nude mice. Tumor size and metastasis were monitored by bioluminescent imaging (Xenogen Corp., Alameda, CA, USA).

### Western blot analysis

Proteins of tissues and cells were dissolved with RIPA buffer (Pioneer, Shanghai, China) containing PMSF (Sigma, Germany). Protein concentration was quantified by BCA protein assay kit (GenStar, Beijing, China). Equal amounts of proteins were separated by 8%–12.5% SDS-PAGE and then transferred to methanol-activated membrane (Roche, Indianapolis, IN, USA), which was incubated with MBD1 (NOVUS, NB100-56537), HDAC3 (Omnimabs, OM256038), DNMT3A (Abways, CY5533), FGFR2 (CST, #23328), BCL-2 (CST, #15071), caspase-3 (Proteintech, 66470-2-Ig), caspase-9 (Proteintech, 66169-1-Ig), MMP2 (Proteintech, 10373-2-AP), MMP9 (Proteintech, 10375-2-AP), vimentin (CST, 5741S), and β-actin antibody (Bioworld Biotechnology, BS6007M) at 4° C overnight. All membranes were washed with TBST and incubated with goat antimouse antibody (Proteintech, SA00001-1) and goat antirabbit antibody (Proteintech, SA00001-2) [33].

### Immunohistochemistry

Immunohistochemistry was used to identify the expression of FGFR2 (Cell Signaling Technology; diluted 1:200), HDAC3 (Omnimabs; diluted 1:200), and MBD1 (NOVUS; diluted 1:200) in the GC and counterpart tissues. Immunohistochemistry was performed as previously described [34].

### Dual luciferase assay

HEK293 cells were seeded into 96-well plates. After 24 h when the cells had 80% confluence, the FGFR2-WT-, MUT-3’-UTR, or empty pmirGLO vectors were cotransfected into HEK293 cells with miR-5701. After 24 h of transfection, Firefly and Renilla luciferase activities were measured using a Dual-Luciferase Assay System (Promega, Fitchburg, WI, USA). Each assay was performed thrice.

### ChIP assay

The binding sites of DNMT3A, MBD1, and HDAC3 to the promoter region of miR-5701 were validated with ChIP analysis. The procedure of ChIP was followed based on the methods as previously described [35]. The primer sequence used for ChIP-PCR are listed in Supplementary Table 2.

### CoIP

Total proteins were extracted after the SGC-7901 cells were harvested, and 10 μg of MBD1, HDAC3 antibody was added into 300 μl of cell lysis for 30 min at 4° C. Then, the proteins were incubated with Dynalmagnetic beads (Invitrogen, Carlsbad, CA, USA) for 2 h and boiled with a loading buffer for 10 min. The supernatant was further applied for Western blot.

### Immunofluorescence microscopy (IF)

SGC-7901 cells were seeded into Nunc Glass Bottom Dishes (Thermo Scientific, Waltham, MA, USA). After 48 h, the transfected SGC-7901 cells were fixed in 4% paraformaldehyde, permeabilized with Triton X-100, and hybridized with the primary antibodies MBD1 (NOVUS; diluted 1:200) and HDAC3 (Omnimabs; diluted 1:200). After hybridization with the secondary antibodies and DAPI, the cells were checked under a fluorescence microscope.

### Statistical analysis

All tests were performed with at least three independent experiments. All data were analyzed with SPSS (SPSS Inc., Chicago, IL, USA) software. Difference between two groups were analyzed by Student’s *t*-test. *P*-values <0.05 were considered as statistically significant.

## Supplementary Material

Supplementary Figure 1

Supplementary Tables
