# Supplementary material for: MBD1/HDAC3-miR-5701-FGFR2 axis promotes the development of gastric cancer
Source: Aging (Albany NY). 2022 Jul 22;14(14):5878–94. doi: 10.18632/aging.204190 (PMC9365560; doi:10.18632/aging.204190)
Supplement: Supplementary Figure 1 [file aging-14-204190-s001.pdf]

SUPPLEMENTARY FIGURE

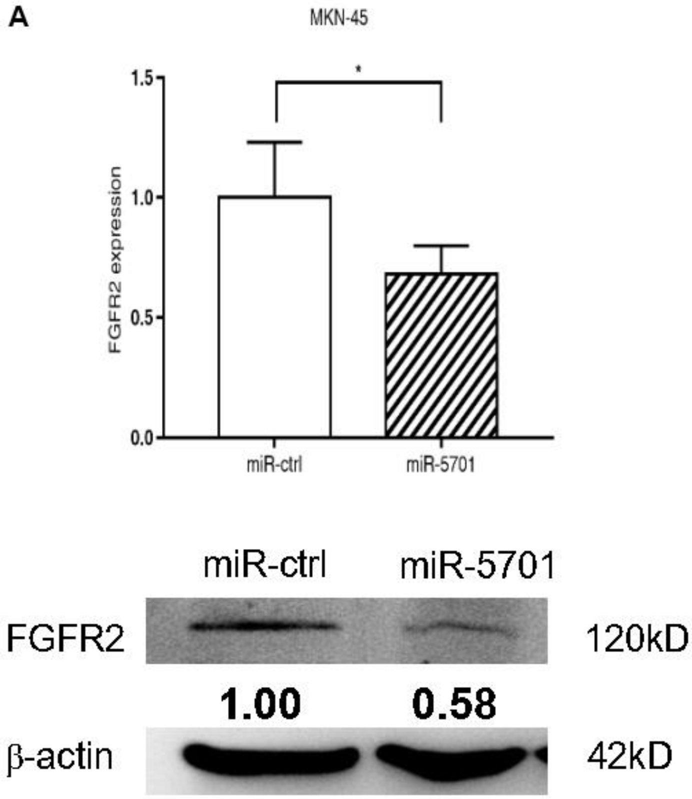

Supplementary Figure 1. FGFR2 was a direct target of miR-5701. (**A**) mRNA level and protein level of FGFR2 after miR-5701 overexpression in MKN-45 cell.
