# Supplementary material for: MBD1/HDAC3-miR-5701-FGFR2 axis promotes the development of gastric cancer
Source: Aging (Albany NY). 2022 Jul 22;14(14):5878–94. doi: 10.18632/aging.204190 (PMC9365560; doi:10.18632/aging.204190)
Supplement: Supplementary Tables [file aging-14-204190-s002.pdf]

SUPPLEMENTARY TABLES

Supplementary Table 1. Primers and oligonucleotides used in this work.

Name	Sequence 5'-3'
miR-5701-inhibitor ctrl	CAGTACTTTTGTGTAGTACAA
miR-5701-inhibitor	AATCAGAACGTGACAATAA
FGFR2 3'UTR-S	CGTTAATTTTTATACTGACAATAAC
FGFR2 3'UTR-AS	TCGAGTTATTGTCAGTATAAAAATTAACGAGCT
FGFR2 3'UTR-MUT-S	CGTTAATTTTTATACTCTCTTTAAC
FGFR2 3'UTR-MUT-AS	TCGAGTTAAAGAGAGTATAAAAATTAACGAGCT
MBD1-S	CCCTGCGTGTGATCTCACC
MBD1-AS	ACCTGACGTTTCCGAGTCTTG
DNMT3A-S	TATTGATGAGCGCACAAGAGAGC
DNMT3A-AS	GGGTGTTCCAGGGTAACATTGAG
DNMT3B-S	GAATTACTCACGCCCCAAGGA
DNMT3B-AS	ACCGTGAGATGTCCCTCTTGTC
DNMT1-S	CCCCTGAGCCCTACCGAAT
DNMT1-AS	CTCGCTGGAGTGGACTTGTG
HDAC3-S	CCTGGCATTGACCCATAGCC
HDAC3-AS	CTCTTGGTGAAGCCTTGCATA
U6-RT	CGCTTCACGAATTTGCGTGTCAT
U6-F	GCTTCGGCAGCACATATACTAAAAT
U6-R	CGCTTCACGAATTTGCGTGTCAT
miR-5701-RT	GTCGTATCCAGTGCGTGTCGTGGAGTCGGCAATTGCACTGGATACGACAATCAGAACG
miR-5701-F	ATCCAGTGCGTGTCGTG
miR-5701-R	TGCTTTATTGTCACGTTCT
GAPDH-F	AGGTCCACCACTGACACGTT
GAPDH-R	GCCTCAAGATCATCAGCAAT
FGFR2-F	TGACATTAACCGTGTTCCTGAG
FGFR2-R	TGGCGAGTCCAAAGTCTGCTAT
siDNMT3A-1-S	GCGUCACACAGAAGCAUAU
siDNMT3A-1-AS	AUAUGCUUCUGUGUGACGC
siDNMT3A-2-S	CGGCUCUUCUUUGAGUUCU
siDNMT3A-2-AS	AGAACUCAAAGAAGAGCCG
siDNMT3A-3-S	CAGUGGUGUGUGUUGAGAA
siDNMT3A-3-AS	UUCUCAACACACACCACUG
siMBD1-1-S	CCGGGAACAGAGAAUGUUU
siMBD1-1-AS	AAACAUUCUCUGUUCCCGG
siMBD1-2-S	GGAGGAGAACAAGGAUGAU
siMBD1-2-AS	AUCAUCCUUGUUCUCCUCC
siMBD1-3-S	GGGAAGAUGGAUGUAAAGU
siMBD1-3-AS	ACUUUACAUCCAUCUUCCC
siHDAC3-1-S	GGGCGGCGGGCGGCGGAGGU
siHDAC3-1-AS	ACCUCCGCCGCCCGCCGCCC
siHDAC3-2-S	GGCTTCACCAAGAGTCTTA
siHDAC3-2-AS	TAAGACTCTTGGTGAAGCC
siHDAC3-3-S	GGAGCAACCCAGCTGAACA
siHDAC3-3-AS	TGTTCAGCTGGGTTGCTCC
Pre-miR-5701-S	AATTCGATTGGACTTTATTGTCACGTTCTGATTGGTTAGCCTAAGACTTGTTCTGATCCAATCAGAACA TGAAAATAACGTCCAATCA
Pre-miR-5701-AS	AGCTTGATTGGACGTTATTTTCATGTTCTGATTGGATCAGAACAAGTCTTAGGCTAACCAATCAGAACG TGACAATAAAGTCCAATCG

Supplementary Table 2. Primers for ChIP-PCR.

Name	Sequence 5'-3'
Primer-1 S	TGTCCCTGGCTTACTTCACT
Primer-1 AS	GTTTGAGGCACATGGATTGA
Primer-2 S	GATTCCATATCTTGGCTATTGTG
Primer-2 AS	TATCCAAAGAAAAGGAAATCAGT
Primer-3 S	AGTAGCCATTCTAAGTGGGGTGA
Primer-3 AS	CATCAGGGAAATGCAAATCAA
Primer-4 S	AGGAAAACATATTTGTTGGTCAT
Primer-4 AS	CAAGGAACTCTTGACATCTCAAT
Primer-5 S	TCTGCCAATTTCTTCCTTTGA
Primer-5 AS	ACTTACGCGATCCTCTTCTGC
Primer-6 S	GGGTACAGAAGAAACATACTGC
Primer-6 AS	ATCATGTTCCAGATCTTAGAGG
Primer-7 S	GCCCACTGTCACCACTGTT
Primer-7 AS	GGGGCTACACTGCTGGTTG
Primer-8 S	CCCACCTCACTGTTGCCA
Primer-8 AS	TATCTGGGGCTGTGCTGC
Primer-9 S	GGGCAGTGACGCCTGAGAT
Primer-9 AS	GTTGGTGGCAGCGATGGA
Primer-10 S	GCTGGCACCTCCTACTCCA
Primer-10 AS	CCTCGTCGGCATCTGACTT
